# Influence of Swimming Training Session on Selected Saliva Components in Youth Swimmers

**DOI:** 10.3389/fphys.2022.869903

**Published:** 2022-04-14

**Authors:** Iwona Grzesiak-Gasek, Urszula Kaczmarek

**Affiliations:** ^1^ Department of Pediatric Dentistry and Preclinical Dentistry, Wroclaw Medical University, Wroclaw, Poland; ^2^ Department of Conservative Dentistry with Endodontics Wroclaw Medical University, Wroclaw, Poland

**Keywords:** swimming training session, youth, salivary components, RPE scale, exercise

## Abstract

Exercise may induce many changes in biochemical parameters of the saliva. Thanks to non-invasive access, saliva can be used as a diagnostic material in physical activity monitoring. The aim of the study was comparison of selected salivary components in swimmers before and after training session. 40 male subjects aged 12–15, out of whom 30 were competitive swimmers and 10 control were involved in the study. Salivary samples were collected from all subjects in the morning, and in the afternoon; from the swimmers, they were also collected before and after the workout. Salivary flow rate-V, pH, total protein-P, alpha-amylase-Amy, salivary peroxidase-SPO, cortisol-C, total antioxidant status-TAS, sialic acid (free-FSA, bound-GSA, total-TSA), calcium-Ca, magnesium-Mg were measured. The swimmers assessed the workout intensity of training session using the RPE Foster’s scale. The circadian rhythm pattern of some salivary components and differences after training were found. In swimmers after the morning exercise significant increase of P (0.83 ± 0.27 vs. 1.10 ± 0.58 g/L), Amy (64.91 ± 70.86 vs. 87.07 ± 92.46IU/L), Ca (3.83 ± 1.33 vs. 4.99 ± 2.24 mg/L), Mg (0.52 ± 0.32 vs. 0.73 ± 0.34 mg/L), TAS (0.64 ± 0.27 vs. 0.72 ± 0.26 mmol/L) and decrease V (0.47 ± 0.37 vs. 0.36 ± 0.22 mg/min), C (5.86 ± 5.00 vs. 3.54 ± 5.07 μg/ml) were found. After the afternoon training significant increase of pH (7.13 ± 0.33 vs. 7.27 ± 0.24), Amy (111.53 ± 120.13 vs. 130.91 ± 161.14IU/L), Ca (3.72 ± 1.34 vs. 4.61 ± 1.58 mg/L), Mg (0.48 ± 0.28 vs. 0.60 ± 0.39 mg/L), TSA (5.64 ± 3.78 vs. 6.10 ± 3.08 mg/L), GSA (3.00 ± 3.06 vs. 3.38 ± 2.26 mg/L), and decrease of V (0.63 ± 0.63 vs. 0.49 ± 0.39 ml/min) were noticed. Before training in the morning in the swimmers significantly higher of V (0.47 ± 0.37 vs. 0.26 ± 0.15 mg/min), TAS (0.64 ± 0.27 vs. 0.40 ± 0.16 mmol/L), and lower pH (7.01 ± 0.46 vs. 7.53 ± 0.33), P (0.83 ± 0.27 vs. 1.86 ± 1.28 g/L), Amy (64.91 ± 70.86 vs. 146.56 ± 114.45IU/L) compared to the control were found. In the afternoon in swimmers before training session significantly lower pH (7.13 ± 0.33 vs. 7.53 ± 0.49) and Amy (111.53 ± 120.13 vs. 170.98 ± 107.72IU/L) in comparison to the control were noticed. The RPE scores were negatively correlated with V (rho = −0.500, *p* = 0.05 and pH (rho = −0.382, *p* = 0.03) measured after the morning session and after the afternoon training with V (rho = −0.570, *p* = 0.01) and Ca (rho = −0.401, *p* = 0.08). The levels of salivary flow rate, alpha amylase, cortisol, calcium, magnesium were associated with swimming training session, and showed circadian variation without a significant effect on their responses to exercise.

## Introduction

Due to the presence of various components synthesized in salivary glands, and originating from the serum and cells, as well as thanks to non-invasive access, saliva can be used as a potential diagnostic material in local and systemic diseases (Mortha et al., 2018; Soares Nunes et al., 2015). Determining the levels of selected salivary components is also used to monitor physical exertion when doing different sports (Chicharro et al., 1998; Diaz et al., 2012; Ligtenberg et al., 2016).

Physical activity activates the autonomic nervous system which impacts the secretion and content of saliva (Ljungberg et al., 1997). The stimulation of the sympathetic system results in the secretion of low volumes of saliva which is high in protein, whereas the stimulation of the parasympathetic system causes increased secretion of water and mucin (Nunes et al., 2015; Ligtenberg et al., 2015). Reduced salivary secretion during intense physical activity results from intensified activity of sympathetic innervation which leads to the constriction of vessels supplying blood to the salivary glands (Ljungberg et al., 1997), as well as from increased vasopressin (antidiuretic hormone) (Walsh et al., 2004), or an increase in salivary viscosity caused by evaporation of water during heavy mouth breathing (Ligtenberg et al., 2016). The increase in the viscosity of saliva observed after exercise can result from several causes (e.g., an increase in concentrations of proteins and mucins due to dehydration with a concomitant decrease in the secretion rate of saliva) (Ligtenberg et al., 2015; Ligtenberg et al., 2016). Exercise might also induce changes in the levels of other salivary components, such as immunoglobulins, hormones, lactate, enzymes and electrolytes (Chicharro et al., 1998; Chicharro et al., 1999; Diaz et al., 2012).

The subjective response of athletes to training and competitions is both dynamic and complex; therefore, it requires the consideration of associated stress.

The objective markers of psychophysiological stress in different sports disciplines that are widely used include the activity of the hypothalamic–pituitary–adrenocortical system (HPA) with the secretion of cortisol, and the sympathetic system, with the secretion of alpha amylase (de Oliveira et al., 2010; Diaz et al., 2013; Jacks et al., 2002).

Some studies showed that salivary alpha-amylase increased more rapidly than salivary cortisol, demonstrating that it is a more immediate indicator of stress than cortisol (Yazdanparast et al., 2009; Crewther et al., 2010; Bruzda-Zwiech et al., 2017). Probably, the difference in response to stress between alpha-amylase and cortisol in the saliva might result from the time latency in the stress response of the sympathetic nervous system and that of the HPA system (Kim et al., 2009).

Physical exercise may lead to an increase in oxidative stress resulting from the production of free radicals and reactive oxygen species (ROS), as well as reduced protective mechanisms that can damage the cells and tissue functions. ROS are neutralized by a complex system of antioxidant defense. However, exercise can lead to imbalance between ROS and antioxidants, i.e., oxidative stress (Urso and Clarkson, 2003). Saliva contains some antioxidant compounds—enzymatic and non-enzymatic, which can decompose different free radicals invading the oral cavity and hydrogen peroxide produced by oral bacteria (Sariri et al., 2013; Damirchi et al., 2015).

Physical exercise may also lead to changes in the content of trace elements and electrolytes in resting mixed saliva (Chicharro et al., 1999; Nabatov et al., 2017). The stimulation of the sympathetic system may result in changes in the salivary flow, reabsorption and the secretion of electrolytes in the secretory cells by modifying the ion concentration of the saliva.

During swimming, similarly to other sport disciplines, the trainee’s goal is to improve their endurance, obtain the desired results and properly manage the risk of potential injuries (Haddad et al., 2017). Training load monitoring during daily training sessions performed with the use of the most common physiological measurements, such as heart rate, blood lactate concentration and oxygen uptake, is a difficult task. As an alternative, to monitor the exercise intensity, the perceived exertion method can be used in which the trainee reports their perception of strenuousness, discomfort, and tiredness (Psycharakis, 2011). Several methods of exertion assessment have been developed. Foster et al. (2001) proposed modified Borg scale based on a 10-point Ratings of Perceived Exertion (RPE) which includes both the intensity and the duration of the training (Haddad et al., 2017). As a tool which measures the exercise intensity, it can also be used to monitor swimming training (Haddad et al., 2017), even in youth athletes performing different types of training session (Lupo et al., 2017).

## Objectives

The aim of the study was to evaluate selected salivary components in swimmers before and after swim training and compare them with sedentary controls and post-workout self-assessment completed by young swimmers. The preliminary hypothesis was that there were differences in the salivary components following training, and the studied salivary variables presented a diurnal variation.

## Materials and Methods

The study included 40 male subjects aged 12 to 15 (mean 13 ± 0.5 years), out of whom 30 were competitive swimmers and the remaining were subjects with sedentary lifestyle (control group, *n* = 10). The inclusion criteria were swimmers recruited from two Sports Championship Clubs in swimming functioning in the city and all of them (*n* = 30) were involved in the study. The control group (*n* = 10) consisted of clinically healthy participants not taking any medicine based on medical interviews who did not engaged in sports competitions, they did sports activity only recreationally and they were in the same range of age and gender as the swimmers. The swimmers trained 21 h per week in the morning (7.00–8.45 a.m.) and in the afternoon (4.00–5.45 p.m.). The swim practice consisted of swimming the distance of 5,000 m. All the recruited participants had to provide a written informed consent from a parent, they had to be willing to undergo salivary sample collection, and to respond to questionnaire items. The subjects who did not meet the inclusion criteria were excluded from the study.

This study protocol has been approved by the Bioethics Committee (No KB-327/2009) of the Medical University of Wroclaw. All research was performed in accordance with relevant guidelines and regulations, and a statement confirming that informed consent was obtained from all participants.

### Salivary Sample Collection and Studied Parameters

Circadian rhythm pattern of the salivary components was taken into consideration and all unstimulated mixed salivary samples were collected from the swimmers in the morning and in the afternoon, before and after the swimming workout. In the test group, salivary samples were taken four times, i.e. in the morning and in the afternoon, before and after swim training. In the control group, saliva was collected twice a day, in the morning and in the afternoon, at the time of the study. The total number of salivary samples collected amounted to 140. During the sample collection, the subjects were sitting with the head bent down and the mouth open. Saliva was taken with a plastic pipette and put into a graded test tube placed on crushed ice. Based on the measurement of the volume of the collected sample, and the time needed to collect it, the salivary flow rate was calculated as ml/min (V). The samples were centrifuged for 10 min at a speed of 3,500 rpm before biochemical assays. The following salivary parameters were assessed: pH (by potentiometric method), total protein—P (by Lowry’s et al. method) (Lowry et al., 1951), alpha-amylase—Amy (by Caraway’s colorimetric method-Alpha Diagnostic Kit), salivary peroxidase - SPO (using Nbs-SCN method) (Mansson-Rahemtulla et al., 1986), salic acid total—TSA, bound—GSA and free - FSA (by periodate-resorcinol method) (Jourdian et al., 1971), cortisol—C (by Elisa Kit from R&D Systems), total antioxidant status—TAS (by TAS assay Randox), calcium—Ca (by method based on formation of chromogenic complex between calcium ions and o-cresolphthaelin using Alpha Diagnostics kit), magnesium—Mg (by colorimetric method based on the reaction of magnesium with Xylidyl Blue-I using Alpha Diagnostics kit).

Moreover, the severity of the workout was self-assessed by the swimmers using Foster’s scale (modified Borg CR-10) which is the category-ratio scale of perceived exertion rating (RPE). The scale is characterized by numerical scores and verbal links (i.e., from 0 -“rest” to 10—“maximal”) referring to the athlete’s perception of efforts (Foster et al., 2001).

The Foster’s RPE scale was administered around 30 min after the end of the training session to assess the internal load of the entire training session. The training load was categorized dichotomical as light (scores 0–4) or high (scores 5–10).

### Statistical Analysis

Depending on the type of variables distribution, Student’s t-test or Mann-Whitney test was used for the independent variables analysis. For the dependent variables, Student’s t-test or non-parametric Wilcoxon’s test was conducted. The Pearson’s correlation coefficient between Foster’s RPE scores and the difference in the salivary parameters before and after the training was calculated.

Bivariate logistic regression analyses for dependent variables in swimmers (light or high training load) according to Foster’s RPE scale and independent variables (salivary parameters) were carried out.

Receiver operating characteristic (ROC) curve analysis was used to evaluate the diagnostic potential of selected salivary constituents to classify the training load. The results were assessed by the area under the ROC curve (AUC) and the cutoff values, which were determined based on the best trade-off between sensitivity and specificity. The level of significance was set at *p* ≤ 0.05. All analyses were computed with Statistica 13.1 software package (StatSoft, Inc., Tulsa, OK, United States).

## Results

### The Time of Day Variations of the Studied Salivary Parameters

Both swimmers before and after training presented the lower levels of flow rate, alpha amylase, TSA, FSA and a higher cortisol in the salivary samples collected in the morning than in the afternoon. Moreover, the swimmers before training in the morning compared with the afternoon hours showed the lower protein content and after training the lower pH and higher magnesium levels. In the control group, we noticed a lower level of SPO, and a higher cortisol content in the morning compared to the afternoon (Table 1).

**TABLE 1:** Concentration of the test components in saliva.

Salivary Parameters	Day time	Non- swimmers (Control group) N = 10, a.m. N = 10, p.m.	Swimmers study group		Before training swimmers vs. non-swimmers	Swimmers before vs. after training
			Before session N = 30, a.m. N = 30, p.m.	After session N = 30, a.m. N = 30, p.m.		
		x ± SD	x ± SD	x ± SD		
Salivary flow rate (V) mg/ml	a.m.	0.26 ± 0.15	0.47 ± 0.37	0.36 ± 0.22	**u = 2.499, *p* = 0.012** ^b^	**z = 2.746, *p* = 0.006** ^c^
	p.m.	0.35 ± 0.19	0.63 ± 0.63	0.49 ± 0.39	u = 1.812, *p* = 0.070^b^	**z = 2.606, *p* = 0.009** ^c^
	a.m. vs. p.m.	z = 1.376, *p* = 0.168^c^	**z = 2.417, *p* = 0.016** ^c^	**z = 2.931, *p* = 0.003**		
pH	a.m.	7.53 ± 0.33	7.01 ± 0.46	7.15 ± 0.33	**t = -3.192 *p* = 0.003** ^a^	t = −1.713 *p* = 0.097^a^
	p.m.	7.53 ± 0.49	7.13 ± 0.33	7.27 ± 0.24	**t = -2.932 *p* = 0.006** ^a^	**t =** −**2.647 *p* = 0.013** ^a^
	a.m. vs. p.m.	t = 0.729, *p* = 0.486^a^	t = −1.59, 7 *p* = 0.121^a^	**t = -2.060 *p* = 0.048** ^a^		
Total protein (P) g/L	a.m.	1.86 ± 1.28	0.83 ± 0.27	1.10 ± 0.58	**u = -2.936, *p* = 0.003** ^b^	**z = 2.919, *p* = 0.003** ^c^
	p.m.	1.40 ± 0.71	1.16 ± 0.46	1.21 ± 0.58	u = −0.687, *p* = 0.492^b^	z = 0.292, *p* = 0.770^c^
	a.m. vs. p.m.	z = 1.376 *p* = 0.169^c^	**z = 4.330 *p* = 0.000** ^c^	z = 0.422 *p* = 0.673^c^		
ά-amylase (Amy) IU/L	a.m.	146.56 ± 114.45	64.91 ± 70.86	87.07 ± 92.46	**u = -1.96, *p* = 0.049** ^b^	**z = 3.610, *p* = 0.001** ^c^
	p.m.	170.98 ± 107.72	111.53 ± 120.13	130.91 ± 161.14	**u = -2.155, *p* = 0.031** ^b^	**z = 2.190, *p* = 0.028** ^c^
	a.m. vs. p.m.	t = 0.562 *p* = 0.508^c^	**z = 3.233 *p* = 0.001** ^c^	**z = 2.049 *p* = 0.040** ^c^		
Calcium (Ca) mg/L	a.m.	3.25 ± 0.79	3.83 ± 1.33	4.99 ± 2.24	t = 1.307, *p* = 0.199^a^	**t = -4.182, *p* = 0.001** ^a^
	p.m.	3.18 ± 0.96	3.72 ± 1.34	4.61 ± 1.58	t = 1.191, *p* = 0.241^a^	**t = -4.439, *p* = 0.001** ^a^
	a.m. vs. p.m.	t = 0.185, *p* = 0.856^a^	t = 0.440, *p* = 0.663^a^	t = 0.890, *p* = 0.376^a^		
Magnesium (Mg) mg/L	a.m.	0.60 ± 0.38	0.52 ± 0.32	0.73 ± 0.34	u = -0.500, *p* = 0.617^b^	**z = 3.548, *p* = 0.001** ^c^
	p.m.	0.44 ± 0.24	0.48 ± 0.28	0.60 ± 0.39	u = 0.359, *p* = 0.719^b^	**z = 1.995, *p* = 0.046** ^c^
	a.m. vs. p.m.	z = 1.376, *p* = 0.168^c^	z = 1.038, *p* = 0.299^c^	**z = 2.130, *p* = 0.033** ^c^		
Total sialic acid (TSA) mg/L	a.m.	3.86 ± 2.84	4.50 ± 2.99	4.70 ± 2.73	u = 0.578, *p* = 0.563^b^	z = 0.956, *p* = 0.333^c^
	p.m.	6.88 ± 6.31	5.64 ± 3.78	6.10 ± 3.08	u = 0.125, *p* = 0.900^b^	**z = 2.151, *p* = 0.031** ^c^
	a.m. vs. p.m.	z = 1.070, *p* = 0.284^c^	**z = 2.595, *p* = 0.009** ^c^	**z = 2.476, *p* = 0.013** ^c^		
Glycosidically bound sialic acid (GSA) mg/L	a.m.	1.78 ± 0.73	2.88 ± 2.03	2.92 ± 1.96	u = 1.437, *p* = 0.151^b^	z = 0.000, *p* = 1.000^c^
	p.m.	3.46 ± 2.64	3.00 ± 3.06	3.38 ± 2.26	u = −0.640, *p* = 0.522^b^	**z = 2.389, *p* = 0.017** ^c^
	a.m. vs. p.m.	z = 1.717, *p* = 0.085^c^	z = 0.148, *p* = 0.882^c^	z = 0.877, *p* = 0.380^c^		
Free sialic acid (FSA) mg/L	a.m.	2.08 ± 2.41	1.78 ± 1.46	1.95 ± 1.37	u = −0.016, *p* = 0.987^b^	z = 1.200, *p* = 0.230^c^
	p.m.	3.41 ± 4.15	2.64 ± 1.76	2.64 ± 1.76	u = 0.734, *p* = 0.463^b^	z = 0.350, *p* = 0.727^c^
	a.m. vs. p.m.	z = 1.172, *p* = 0.241^c^	**z = 3.08,1 *p* = 0.002** ^c^	**z = 2.252, *p* = 0.024** ^c^		
Total Antioxidant Status (TAS) mmol/L	a.m.	0.40 ± 0.16	0.64 ± 0.27	0.72 ± 0.26	**t = 2.666, *p* = 0.011** ^a^	**t = -2.914, *p* = 0.007** ^a^
	p.m.	0.59 ± 0.41	0.55 ± 0.24	0.61 ± 0.29	t = −0.341, *p* = 0.735^a^	t = −.609, *p* = 0.118^a^
	a.m. vs. p.m.	t = −0.210, *p* = 0.257^a^	t = 1.356, *p* = 0.186^a^	t = 1.783, *p* = 0.085^a^		
Salivary peroxidase (SPO) mIU/ml	a.m.	1.31 ± 0.63	1.26 ± 0.99	1.18 ± 0.93	u = −0.633, *p* = 0.526^b^	z = 0.05, *p* = 0.959^c^
	p.m.	1.56 ± 0.60	1.60 ± 1.47	1.73 ± 1.65	u = −0.968, *p* = 0.333^b^	z = 1.121, *p* = 0.262^c^
	a.m. vs. p.m.	**z = 2.073, *p* = 0.038** ^c^	z = 1.059, *p* = 0.289^c^	z = 1.676, *p* = 0.094^c^		
Cortisol (C) μg/ml	a.m.	6.43 ± 3.94	5.86 ± 5.00	3.54 ± 5.07	u = −0.999, *p* = 0.317^b^	**z = 2.99, *p* = 0.003** ^c^
	p.m.	4.18 ± 4.34	2.21 ± 2.85	2.20 ± 2.67	u = −1.843, *p* = 0.065^b^	z = 0.545, *p* = 0.586^c^
	a.m. vs. p.m.	**z = 2.395, *p* = 0.016** ^c^	**z = 4.165, *p* = 0.0001** ^c^	**z = 2.520, *p* = 0.012** ^c^		

a t-student test

b U-Mann-Whitney test

c Wilcoxon test.

The bold values indicates that values are significance (*p* ≤ 0.05).

### Levels of the Salivary Parameters Before and After Training Session and in the Control Group

**FIGURE 1:**
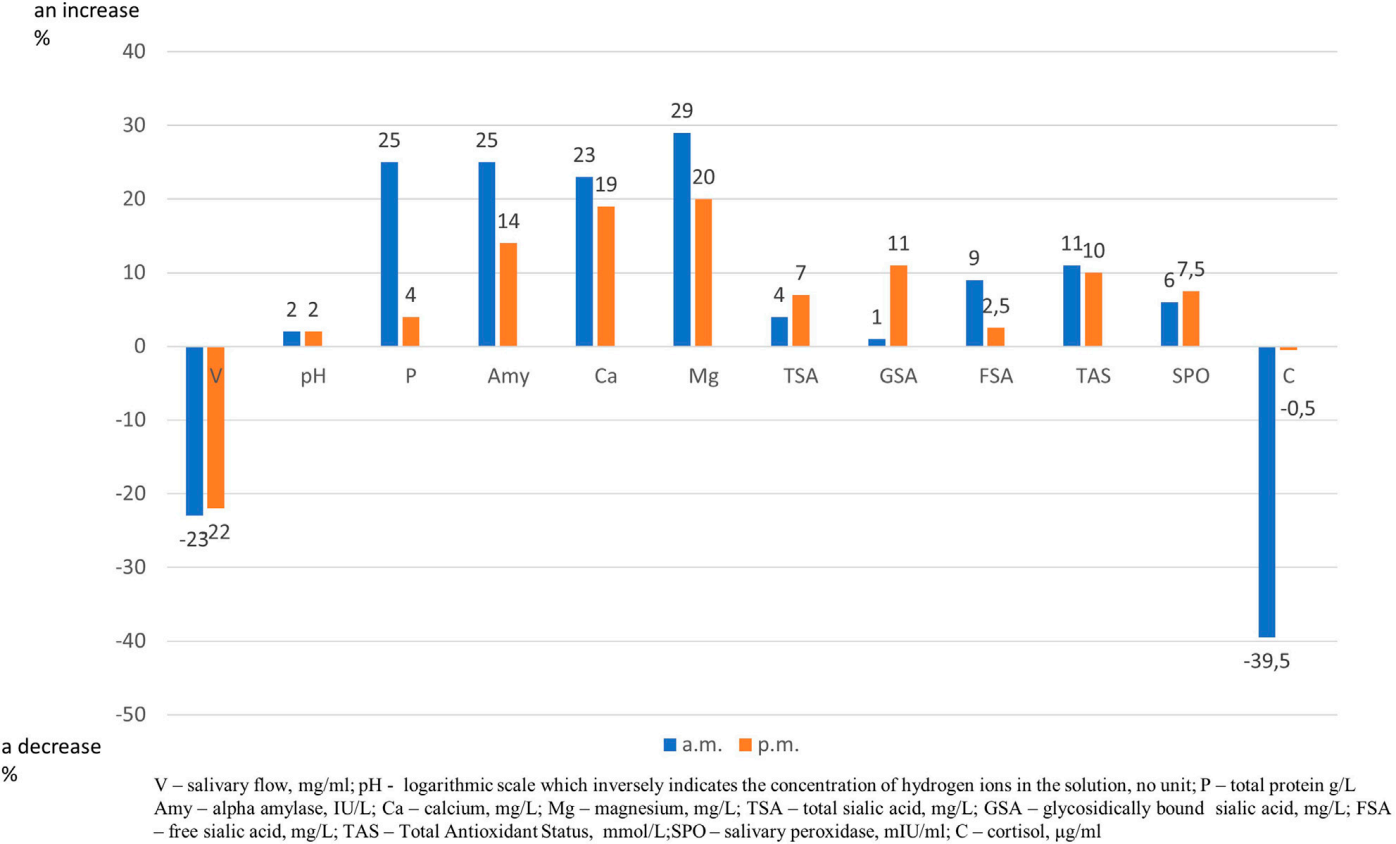
Effect of trainning session on relative changes in salivary parameters. “Zero” is set as 100% level of salivary parameters before trainning session and an increase or a decrease is expressed as percentage.

After training both in the morning and in the afternoon a decrease in salivary flow rate and an increase in alpha amylase, calcium and magnesium content were found. Moreover, after morning training, an increase in protein and TAS levels and a decrease of cortisol content, and after afternoon training an increase in TSA and GSA contents were noticed (Table 1). The effect of exercise on relative changes in the concentration of salivary components, presented as 100% baseline value, is illustrated in Figure 1.

Comparing salivary components in swimmers before training session with the control group in the morning and in the afternoon lower pH and alpha amylase levels in saliva were found in the controls. Moreover, in the morning hours, higher salivary flow rate, lower protein and higher TAS levels were observed in swimmers in comparison to the control group (Table 1).

### Salivary Component Output Before and After Training in Swimmers

After the morning training session, a slight increase in the output was observed for all tested salivary components except for the cortisol, and it was statistically significant for total protein, alpha amylase, calcium, magnesium, TSA, FSA and TAS.

**FIGURE 2:**
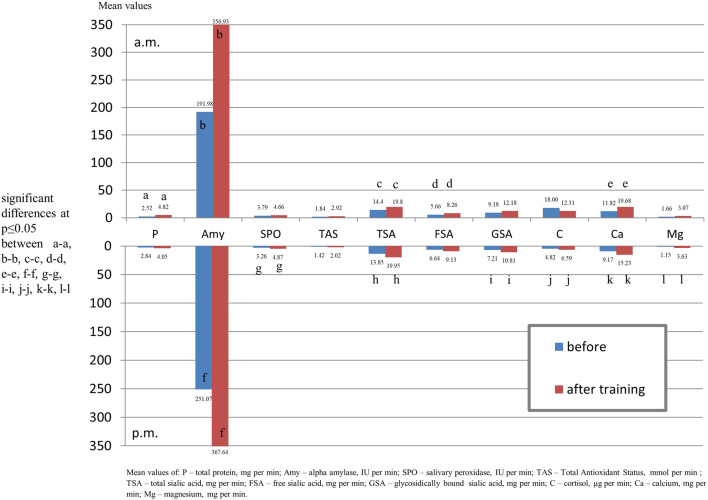
Output of salivary components (mg/µg/U per min.) of youth swimmers before training and youth non-swimmers in the morning and afternoon hours.

Following the afternoon training session, a slight increase in the output was observed for all salivary components. A significant increase in the output was found for alpha-amylase, calcium, magnesium, TSA,GSA, TAS, salivary peroxidase and cortisol (Figure 2).

**FIGURE 3:**
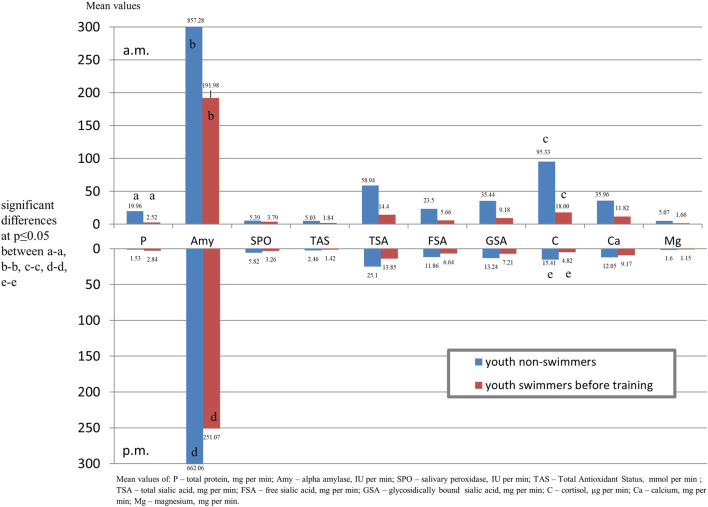
Output of salivary components (mg/µg/U per min.) of youth swimmers before and after training in the morning and afternoon hours.

Comparing salivary components output in swimmers before training with the control group in the morning, significantly lower levels of protein and alpha amylase and in the afternoon lover content of alpha amylase and cortisol in the swimmers were found (Figure 3).

### Assessment of the Perceived Exertion of Training Session

**FIGURE 4:**
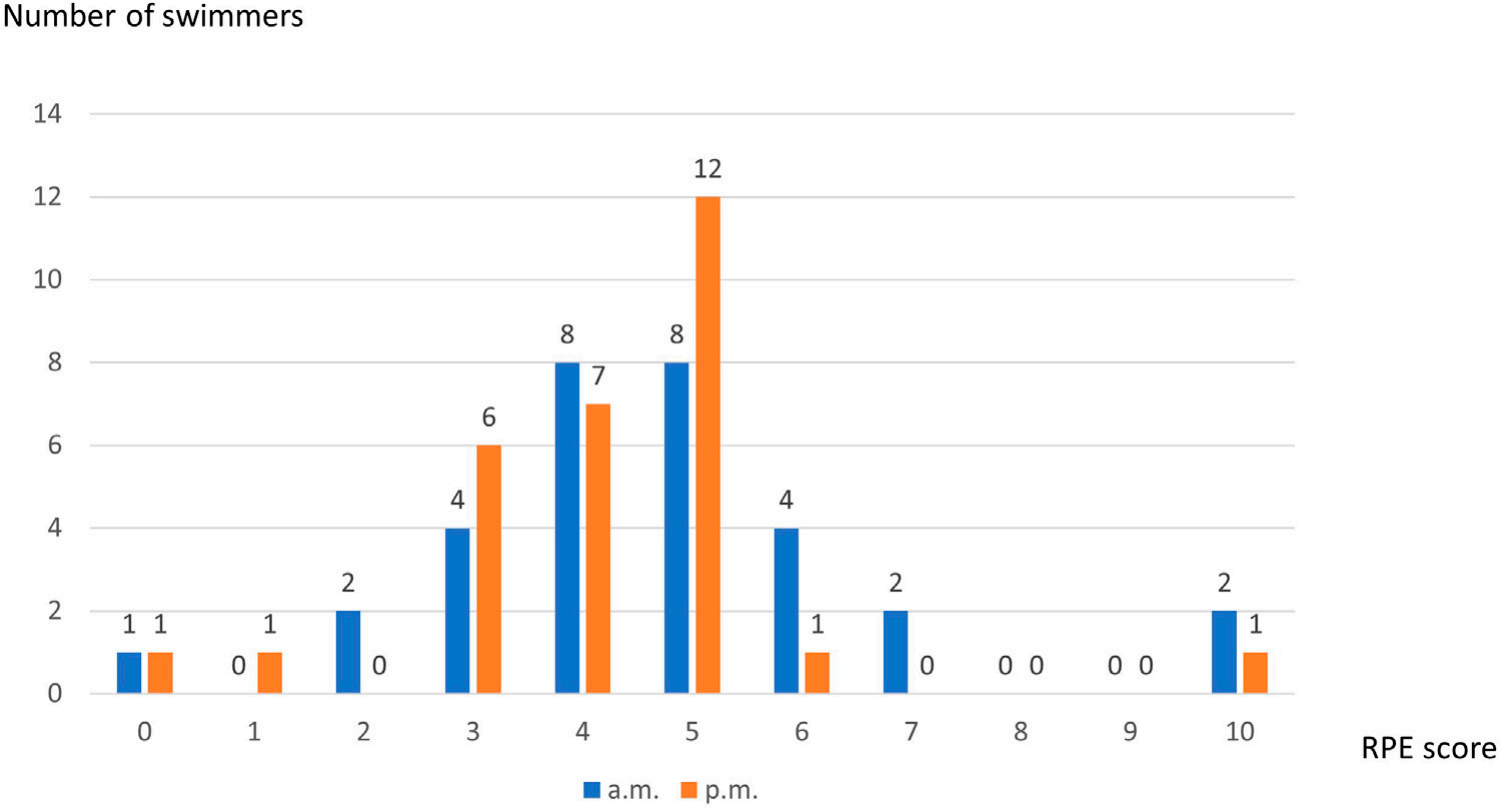
Distribution of RPE scores after swimming session in the morning and in the afternoon.

Mean value of Foster’s RPE scale was 4.53 ± 1.83 after morning training session with the most responses “somehow hard” and “hard (8 subjects each) and 4.43 ± 1.98 following the afternoon session with the most self-asssessment “somehow hard” (*n* = 7) and “hard” (*n* = 12). Distribution of RPE scores was presented in Figure 4.

### Correlation Between Changes in the Salivary Parameter Levels due to Training Session and Perceived Exertion

Correlation between the differences in the pre- and post-training session concentration of salivary parameters, and Foster’s RPE scores only showed a significant negative correlation with salivary flow rate and pH (rho = −0.500, *p* = 0.005 and rho = −0.383, *p* = 0.040, respectively) in the morning. In the afternoon, the correlation between the differences in the pre- and post-training concentration of salivary parameters and Foster’s RPE scores only showed a significant negative correlation with salivary flow rate and calcium (rho = −0.571, *p* = 0.001 and rho = −0.401, *p* = 0.028, respectively) (Table 2).

**TABLE 2:** Correlation between RPE values and salivary parameters in swimmers after the morning and the afternoon training sessions.

Salivary Parameters	Time of the day			
	Morning		Afternoon	
	Rho value	*p* value	Rho value	*p* value
Flow rate	−**0.500**	**0.005**	−**0.570**	**0.001**
pH	−**0.382**	**0.037**	−0.147	0.436
Protein	0.021	0.923	−0.086	0.651
Amy	0.018	0.923	0.082	0.664
Calcium	0.097	0.607	−**0.401**	**0.028**
Magnesium	−0.119	0.529	−0.144	0.446
TSA	0.139	0.463	0.233	0.215
GSA	−0.071	0.706	0.193	0.306
FSA	0.348	0.059	0.064	0.734
TAS	−0.081	0.671	0.049	0.796
SPO	−0.051	0.785	−0.088	0.644
Cortisol	0.047	0.803	0.325	0.080

The bold values indicates that values are significance (*p* ≤ 0.05).

**FIGURE 5:**
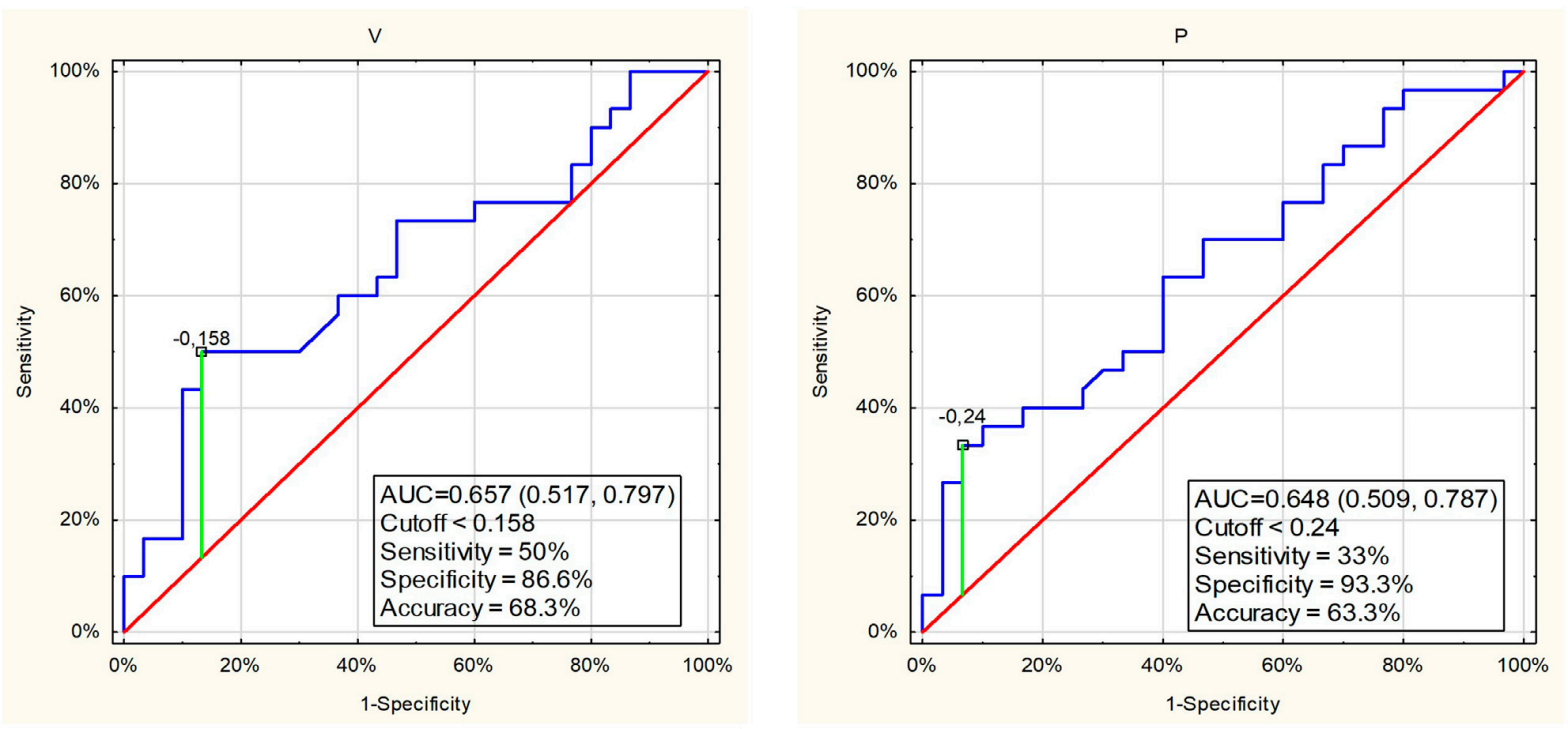
ROC curves and cutoff values for salivary parameters those showed significant difference between the swimmers with easy or hard perceived training session load.

Logistic regression revealed that out of separately analyzed salivary variables, only salivary flow rate and protein content were associated with the perceived training load (Table 3). However, ROC curve analysis indicated that the salivary flow rate and protein concentration were weak classifiers to discriminate the perceived training load of swimmers. The specificity for these variables was high but sensitivity low, and the AUC were 0.657 and 0.648 (Figure 5). Therefore, salivary flow rate and protein content turned out to be weak markers to distinguish the swimmers with light and high training session load.

**TABLE 3:** Logistic regression analysis for self-assessment of easy or hard training session as dependent variables.

Results of bivariate logistic regression for dependent variable of self-assessment easy or hard training session					
Salivary parameters (independent variables included into regression model)	b	OR	lower CI 95%	upper CI 95%	*p*
Flow rate	0.422	**0.001**	0.000013	0.127197	**0.004**
pH	−6.647	0.329	0.043111	2.525735	0.285
Protein	−1.108	**0.069**	0.008525	0.559017	**0.012**
Calcium	−2.673	0.746	0.436093	1.277523	0.286
Magnesium	−0.292	0.171	0.013808	2.120831	0.169
GSA	−1.765	0.762	0.493788	1.177044	0.220
TAS	−0.713	3.911	0.086211	177.4498	0.483

b, logistic regression coefficient; OR, odds ratio p- value level of statistical significance; CI, confidence interval (at the level 95%).

The bold values indicates that values are significance (*p* ≤ 0.05).

## Discussion

The preliminary hypothesis that were differences in the salivary components following training session, and the studied salivary variables presented a diurnal variation was supported. Biological rhythm patterns have been demonstrated in salivary flow rate and in the content of some salivary components; therefore, we have taken into account this phenomenon. Our data presented the time of day variations in the levels of some studied salivary parameters. In all groups, we observed a lower salivary flow rate in the morning than in the afternoon similarly to other studies (Dimitriou et al., 2002; Shirakawa et al., 2004). It is explained by a higher activity of the sympathetic nervous system in the morning hours than at other times of the day.

We also noticed the diurnal course of salivary alpha amylase, i.e., lower in the morning in comparison to the afternoon, which was consistent with the data obtained by Nater et al. (2007) and Pajcin et al. (2019). However, the circadian rhythm fluctuations in salivary amylase activity are much smaller than the stress-induced variations; therefore, salivary amylase activity is a valuable indicator of the changes in sympathetic nervous activity (Yamaguchi et al., 2006). Circadian rhythm of cortisol level in the saliva has been described in many studies (Shirakawa et al., 2004; Asok et al., 20016; Ushiki et al., 2020). Our data confirmed this relationship, as we found a higher cortisol level in the morning in comparison to the afternoon in all groups of subjects. However, Dimitrou et al. (2002) did not observe any significant interaction effect on salivary cortisol between the time of day and exercise. Sialic acid in the saliva also presented some diurnal fluctuation, i.e., a lower content in the morning than in the afternoon, which was consistent with Hodosy and Celec’s data (2005).

Physical exercise leads to changes in some biochemical components of blood, which are mirrored in the saliva. Some diminishing of the salivary flow rate after swimming training observed in our study is consistent with the previous findings after a swimming session (Dimitriou et al., 2002; Bretz and Carrilho, 2013) or other physical exercise (Mulic et al., 2012). Moreover, other studies presented a gradual decrease in the salivary flow rate in swimmers undergoing repetitive altitude training (Watanabe et al., 2019), or no difference in salivary flow rate throughout the training season of elite swimmers (Díaz Gómez et al., 2013). The decrease in salivary flow has been explained by an increase in sympathetic activity during intense exercise, since sympathetic innervations cause a marked vasoconstriction, resulting in reduced salivary volume (Chicharro et al., 1998). It can also be a consequence of sweat-induced dehydration and restricted fluid intake during exercise. However, in our study the swimmers were allowed to drink energy drinks before training and the heat produced by the bodies was released directly to the water the temperature of which ranged from 26 to 28°C. Therefore, they were not exposed to dehydration. A tendency for a decrease in average salivary pH after the swimming session has been reported (Bretz and Carrilho, 2013). However, other data pointed to an opposite trend similarly to other studies (Ligtenberg et al., 2015).

The assessment of salivary total protein content has been proposed as a prospective marker to dehydration after exercise following an increase in β-sympathetic activity in salivary glands (Diaz et al., 2012). Some increase in total salivary protein content after aerobic exercise on cycle-ergometers was observed (Ligtenberg et al., 2016). Similarly our data showed a significant increase in protein after the morning training.

It has been suggested that an increase in alpha amylase secretion is responsible for an increase in the total salivary protein content; therefore, monitoring salivary protein can be an alternative noninvasive biochemical measure for determining exercise intensity (de Oliveira et al., 2010). Alpha amylase being one of the most abundant components in human saliva plays an important role in the oral cavity by the initial enzymatic digestion of dietary starch and providing carbohydrate nutrition for oral bacteria, binding to a selected group of oral bacteria to teeth and a component of the dental pellicle (Nikitkova et al., 2013). The levels of salivary alpha amylase undergo changes due to physical and acute psychological stressors. Therefore, they can be considered a marker of sympathetic activation. The association of the enzyme level following exercise has been extensively investigated. An increase in this enzyme in response to stress related to physical exercise, such as treadmill exercise, running, basketball, swimming, taekwondo was reported (de Oliveira et al., 2010; Diaz et al., 2013; Edmonds et al., 2015). Our data confirmed the previous results, as we found a higher level of the enzyme following swimming training. The elevated enzyme level in the saliva is a fast response to stress situations, and it returns to the baseline level at about 30 min post exercise (Kennedy et al., 2001). Diaz et al. (2013) noticed some variation of salivary amylase during the 21-week training season in swimmers (the decline up to the middle of the season with following increase by the end) and a correlation with the intensity and load of training. Edmonson et al. (2015) examining elite swimmers with disability during a 14-week swimming program found that salivary amylase was elevated at the beginning of week 7 in comparison to the baseline. Sinnott-O'Connor et al. (2018) observed a significant increase in salivary alpha amylase during intensified training concurrent with an increase in the training load during training and competition in Paralympic swimmers. Our study has not found any correlation between salivary amylase and the RPE value like in participants in a maximal treadmill test (Gallina et al., 2011). Some possible explanation might be underestimation of load of training by individuals.

Cortisol is a well-known biomarker related to physical training and psychological stress, which is produced in response to a stressor by activation of the hypothalamic-pituitary-adrenal (HPA) axis. Changes in the cortisol level in the blood are reflected in its content in saliva. Salivary cortisol is unconjugated and independent on salivary flow rate while serum cortisol is a steroid conjugated to corticosteroid-binding globulin. However, the observed changes in salivary cortisol depend on the size and duration of the type of physical exercise and duration (Anderson and Wideman, 2017). Jacks et al. (2002) found that only exercise of high intensity and long duration results in significant elevations of salivary cortisol. Majumdar and al. (2019) noticed that the cortisol fluctuates with the intensity and duration of the exercise as they found no significant difference in cortisol level in the plasma in swimmers being in the preparatory (base training period) and pre competitive phase, but a significantly higher increase between swimmers in the competitive phase compared to the preparatory phase (Majumdar et al., 2019). Bruzda-Zwiech et al. (2017) observed a decrease in salivary cortisol after basketball or volleyball training in young males. Klentrou et al. (2016) also noticed a lowered salivary cortisol in prepubertal boys following resistance and plyometric exercise protocols. Yazdanparast et al. (2009) examined the effect of different exercise intensity (low, medium and high) on the salivary concentration of cortisol in young female swimmers, and they found no significant correlation between cortisol after exercise with high and low intensity. Our data showed lowering of salivary cortisol after swimming training which is consistent with the abovementioned results. However, Dimitrou et al. (2002) revealed a significant increase in salivary cortisol after swimming exercise and no significant time of day variation in contrast to our data. We did not find any significant correlation between salivary cortisol levels measured after training with RPE scale. McGuigan et al. (2004) found that the low intensity exercise showed lower RPE values and lower cortisol level in saliva compared to the high intensity exercise. Maya et al. (2016) observed correlation of salivary cortisol with RPE value only after final football match that caused over 50% increase of cortisol and lack of the relationship when the cortisol increase was about 25%. This findings could explain our results as after the morning swimming session we observed a decrease of salivary cortisol (ca. 39%) and after afternoon session no change.

Rapid breathing during physical activity increases the oxygen demand as a result of boosted metabolism which leads to increased production of harmful reactive oxygen species (ROS) and free radicals. Their negative effects can be neutralized by antioxidants. Saliva contains many antioxidant compounds, which comprise enzymes (e.g., peroxidase) and small molecules (e.g., ascorbic acid, sialic acid). Salivary peroxidase is produced by the acinar cells of salivary glands and catalyzes the oxidation of thiocyanate ions by hydrogen peroxide, thus preventing toxic accumulation of H_2_O_2_ (Battino et al., 2002). Sariri et al. (2013) and Damirchi et al. (2015) noticed an increase in the salivary peroxidase activity in athletes immediately after an intense run on the treadmill, which returned to the basal value one hour after an exercise. However, we did not find any significant difference in the level of SPO after swimming training.

Free sialic acid (FSA) present in the saliva is considered as one of the biomarkers of some diseases. Cavas et al. (2005) found a significant increase in free sialic acid in the saliva in Judo athletes after a two-hour-long training session, and they suggested sialic acid as an alternative marker of oxidative stress. However, our data did not show such a relationship because no significant increase in the levels of free sialic acid levels was identified after swimming training.

Our data revealed a significant increase in calcium and magnesium concentration after the swim training. The results were partially consistent with the findings of Chicharro et al. (1999), as they showed a slight decrease in calcium content and a significant increase in magnesium concentration in the mixed saliva following maximal exercise on cycloergometer. Moreover, Bretz and Carrilho (2013) presented a significant increase in calcium in the saliva in male competitive swimmers exposed to 2-h swimming training in a gas-chlorinated swimming-pool.

Despite the significant differences in mean value of the most studied variables after swimming training, salivary rate and total protein levels after ROC curve analysis indicated weak discriminatory power of these variables for the differentiation of children with light and high perceived load of swimmer training.

This study has some limitations. We assessed only selected salivary parameters and thus could not fully characterize all potential changes in the salivary components. Moreover, we did not compare the same studied components in the saliva and the blood. The strength of the study was that we took into consideration circadian rhythm of the studied salivary parameters.

Within the limitations of the study, it may be stated that salivary flow rate, the levels of alpha amylase, cortisol, calcium and magnesium were associated with swimming training and all variables showed a circadian variation without a significant effect on their responses to exercise.

## Data Availability

The raw data supporting the conclusion of this article will be made available by the authors, without undue reservation.
